# Commentary: Low-dose ionizing radiation and the exposure–lag response: protocol for a prospective cohort study on The Health Effects of Chongqing Occupational Radiation Workers

**DOI:** 10.3389/fpubh.2025.1716410

**Published:** 2025-12-16

**Authors:** Colin R. Muirhead

**Affiliations:** Independent Statistician and Epidemiologist, Newcastle upon Tyne, United Kingdom

**Keywords:** epidemiological studies, radiation workers, study protocols, radiation risks, cohort studies

There is a large literature on epidemiological studies of radiation workers, with hundreds of studies published over the past century, dating back to the first studies of radiologists (1). More recent studies have looked at the health of workers in the nuclear industry, such as the INWORKS (2) and Mayak worker studies (3, 4), as well as workers in medical professions [e.g., (5)] and other occupations (1). Some of these studies were based around protocols that were published at the outset of the research, for example, a prospective cohort study of radiation workers in Korea (6). There are many benefits to researchers, funders, and especially the workers themselves of having a clear idea at the outset of what the proposed study involves. Consequently, protocols such as those of Qin et al. (7) for their study of radiation workers in Chongqing (China) are to be welcomed. Nevertheless, there are some important points to bear in mind when preparing these protocols.

First, it is valuable to refer to internationally agreed guidelines for standard protocol items in observational studies. As part of the EQUATOR network for enhancing the quality and transparency of health research (https://www.equator-network.org/), the SPIROS guidelines aim to facilitate the drafting of high-quality observational study protocols (https://www.maastrichtuniversity.nl/spiros-2024-responsible-reporting). Complying with guidelines of this type might be seen by some as simply a “tick-box” enterprise. I disagree; I believe that these guidelines can help researchers think more deeply about some critical aspects of study design. Qin et al. have addressed many of the standard protocol items on the SPIROS checklist in their protocol, including the study design and setting, inclusion and exclusion criteria, a sample size calculation, and ethical approvals and consent. However, it is unclear how potential confounding will be addressed. For example, when assessing possible associations between radiation exposure and hearing loss, workers' exposure to noise would be a key consideration. If information were not collected on such an important cause of hearing loss, then it would be difficult to place much credence on any association with radiation exposure. Similarly, I would have found it helpful to see details of Qin et al.'s plans for database management and quality control and for dealing with loss to follow-up in statistical analyses.

My second point relates to a feasibility study that might be performed before finalizing the study protocol. Qin et al. devote a substantial part of their article to what they describe as “the study population in 2016.” However, it's unclear whether these workers formed the basis of a feasibility study or whether they will be included in the full study, given that study recruitment is due to take place from 2024 to 2028. If these earlier workers are not to be included in the study, then it would have been valuable—as in any feasibility study—to consider whether these workers are likely to have similar characteristics to workers in the full study. If this were not the case, then the relevance of the feasibility study might well be very limited.

Third, there needs to be a clear and robust basis for any sample size calculation. Sadly, I believe that this is a key deficiency of Qin et al.'s protocol. They have based their sample size calculation on the “average white blood cell count for the population in the Chinese radiation group,” based on a meta-analysis of a few hundred Chinese radiation workers (8). However, there is no discussion whether the radiation doses received by these workers would be similar to those received by future workers in the Chongqing study. In addition, Qin et al.'s calculation is based on simply looking for a difference between groups in white blood cells, whereas their aim is to examine exposure–lag relationships. This is a much more complex task, as demonstrated—for example—in an analysis of German uranium miners (9) and in INWORKS (2), and would undoubtedly require a population of at least a few thousand workers if the radiation exposures were substantial (as in the German uranium miner study) or even hundreds of thousands of workers (as in INWORKS) if radiation doses were generally low. Furthermore, it appears that blood cell counts would be assessed only every 2 years in the Chongqing study, in which case it would not be possible to detect any short-term variation in white blood cell counts.

This highlights a wider issue: the importance when designing a study protocol of taking full account of the earlier literature. Reports by international bodies such as the United Nations Scientific Committee on Ionising Radiation (UNSCEAR) have highlighted how key methodological issues such as bias, confounding, and low statistical power can affect epidemiological studies of populations exposed to ionizing radiation (10). As I mentioned previously, there have been many studies of radiation workers in different countries, and the most informative of these are based on cohorts of thousands of workers at the very least, if not more. There is a good reason for this: in order to assess the impacts of low-dose occupational radiation exposure on chronic diseases such as cancer or circulatory disease, very large cohorts are needed. For example, in the INWORKS study of over 300,000 workers followed for nearly 35 years on average and with an average individual cumulative dose of 17.7 mGy, the 90% confidence interval for the excess relative risk for solid cancer mortality was 27–77% per Gy (lagged by 10 years). Therefore, even with the long follow-up of this very large cohort, the upper and lower limits of the confidence interval differed by roughly a factor of 3. Qin et al. do not plan to study cancer, but rather examine other outcomes, such as white blood cell count, thyroid hormones, skin injuries, and hearing loss. Nevertheless, for a cohort of just over 800 radiation workers with just a few years' follow-up and an average annual dose of approximately 0.5 mSv, it is clear that the upper and lower confidence limits for radiation risk estimates derived from the Chongqing study would differ by much more than a factor of 3. Furthermore, the International Commission on Radiological Protection has assessed that workers receiving less than 20 mSv per year would be unlikely to receive skin injuries or incur hearing loss as a consequence of their radiation exposure (11).

Qin et al. are to be congratulated for publishing the protocol for their study. However, it is vitally important to take adequate account of internationally agreed guidelines and findings from previous research when designing a study such as this. In this way, epidemiological studies can play a valuable role in protecting the health of workers.

## References

[1] Wakeford R. Radiation in the workplace—a review of studies of the risks of occupational exposure to ionising radiation. J Radiol Prot. (2009) 29:A61. doi: 10.1088/0952-4746/29/2A/S05 19454806

[2] Richardson DB Leuraud K Laurier D Gillies M Haylock R Kelly-Reif K . Cancer mortality after low dose exposure to ionising radiation in workers in France, the United Kingdom, and the United States (INWORKS): cohort study. BMJ. (2023) 382:e074520. doi: 10.1136/bmj-2022-074520 37586731 PMC10427997

[3] Sokolnikov ME Stram DO Preston DL Sosnina SF Tsareva YV Moroz BE . Leukemia, lymphoma, and multiple myeloma mortality in the Russian Mayak Worker Cohort 1948-2015. Radiat Res. (2025). doi: 10.1667/RADE-23-00059.1 41125086

[4] Azizova TV Grigoryeva ES Hamada N. Dose rate effect on mortality from ischemic heart disease in the cohort of Russian Mayak Production Association workers. Sci Rep. (2023) 13:1926. doi: 10.1038/s41598-023-28954-w 36732598 PMC9895442

[5] Linet MS Little MP Kitahara CM Cahoon EK Doody MM Simon SL . Occupational radiation and haematopoietic malignancy mortality in the retrospective cohort study of US radiologic technologists, 1983-2012. Occup Environ Med. (2020) 77:822–31. doi: 10.1136/oemed-2019-106346 32967989 PMC8527846

[6] Seo S Lim WY Lee DN Kim JU Cha ES Bang YJ . Assessing the health effects associated with occupational radiation exposure in Korean radiation workers: protocol for a prospective cohort study. BMJ Open. (2018) 8:e017359. doi: 10.1136/bmjopen-2017-017359 29602835 PMC5884371

[7] Qin X-L Huang Q Zhang H-W Zeng Y Lin X-S Fan X-Y . and Xia Y-Y. Low-dose ionizing radiation and the exposure–lag response: protocol for a prospective cohort study on the Health Effects of Chongqing Occupational Radiation Workers. Front Public Health. (2025) 13:1531546. doi: 10.3389/fpubh.2025.1531546 39931302 PMC11808129

[8] Zhang X Zhao Y. Meta analysis of the effect of low dose ionizing radiation on peripheral blood cells of radiation workers. Chin J Radiol Health. (2016) 25:406–9.

[9] Aßenmacher M Kaiser JC Zaballa I Gasparrini A Küchenhoff H. Exposure-lag-response associations between lung cancer mortality and radon exposure in German uranium miners. Radiat Environ Biophys. (2019) 58:321–36. doi: 10.1007/s00411-019-00800-6 31218403

[10] United Nations Scientific Committee on the Effects of Atomic Radiation (UNSCEAR). Sources, Effects and Risks of Ionizing Radiation. UNSCEAR 2017 Report to the General Assembly. Scientific Annex A. Principles and Criteria for Ensuring the Quality of the Committee's Reviews of Epidemiological Studies of Radiation Exposure. New York, NY: United Nations (2017). p. 19–64. E.18.IX.1.

[11] Stewart FA Akleyev AV Hauer-Jensen M Hendry JH Kleiman NJ Macvittie TJ . Publication 118: ICRP statement on tissue reactions and early and late effects of radiation in normal tissues and organs–threshold doses for tissue reactions in a radiation protection context. Ann ICRP. (2012) 41:1–322. doi: 10.1016/j.icrp.2012.02.001 22925378

